# Exposome-Explorer 2.0: an update incorporating candidate dietary biomarkers and dietary associations with cancer risk

**DOI:** 10.1093/nar/gkz1009

**Published:** 2019-11-14

**Authors:** Vanessa Neveu, Geneviève Nicolas, Reza M Salek, David S Wishart, Augustin Scalbert

**Affiliations:** 1 International Agency for Research on Cancer (IARC), Nutrition and Metabolism Section, Biomarkers Group, 150 Cours Albert Thomas, F-69372 Lyon Cedex 08, France; 2 Department of Computing Science, University of Alberta, Edmonton, AB T6G 2E8, Canada

## Abstract

Exposome-Explorer (http://exposome-explorer.iarc.fr) is a database of dietary and pollutant biomarkers measured in population studies. In its first release, Exposome-Explorer contained comprehensive information on 692 biomarkers of dietary and pollution exposures extracted from the analysis of 480 peer-reviewed publications. Today, Exposome-Explorer has been further expanded and contains a total of 908 biomarkers. Two additional types of information have been collected. First, 185 candidate dietary biomarkers having 403 associations with food intake (as measured by metabolomic studies) have been identified and added. Second, 1356 associations between dietary biomarkers and cancer risk in epidemiological studies, which were collected from 313 publications, have also been added to the database. Classifications for both foods and compounds have been revised, and new classifications for biospecimens, analytical methods and cancers have been implemented. Finally, the web interface has been redesigned to significantly improve the user experience.

## INTRODUCTION

Cancer is a multifactorial group of diseases caused by multiple genetic and environmental factors. Understanding the links between environment and cancer risk in populations as well as accurately estimating and describing population exposures to different cancer risks factors are among the central objectives of cancer epidemiology (1). In epidemiological studies, population-level dietary exposures are traditionally measured using food frequency questionnaires, food records or food recalls. These food survey methods have led to important discoveries about cancer etiology (2,3). However, the development of high throughput technologies such as metabolomics—which is able to detect thousands of chemicals in a single biological sample—has ‘raised the bar’ with regard to objectively measuring cancer-associated food and other environmental exposures. Indeed, the idea of a cancer ‘exposome’ is gaining increasing traction among cancer epidemiologists. The exposome refers to all external agents to which the human body is exposed, from conception onward. Key to exposome studies is the assumption that specific chemical biomarkers can act as surrogates of exposure that provide more direct, unbiased and objective measures of environmental exposures than possible with traditional methods (4,5).

The Exposome-Explorer database was started in 2012 and its website officially launched in 2017 (6). The goal of Exposome-Explorer is to compile all available information on exposure biomarkers described in the scientific literature. The textual information found in scientific articles represents an important source of data but is intrinsically difficult to evaluate and interpret. The objective of Exposome-Explorer is to identify biomarker information from peer-reviewed scientific publications, collect it, store it, and organize it, and finally make it more easily accessible to researchers. In the first release of Exposome-Explorer, data from 480 publications was manually collected for 692 dietary and pollutant biomarkers. This data included biomarker concentrations in biospecimens for different populations, temporal reproducibility, and correlations values between biomarkers and exposures. Chemical information, analytical methods, biospecimens and populations were also documented. Data was collected both for dietary and pollutant biomarkers, covering a wide range of dietary and chemical exposures.

For the second release of Exposome-Explorer, we have placed an increased focus on dietary biomarkers. In this release, the addition of two new types of dietary exposure information as well as important changes to the web interface are described. Firstly, a large number of candidate dietary biomarkers previously identified via metabolomics studies have been added. In particular, a number of novel biomarkers of exposure have been identified by comparing metabolomic profiles between exposed and non-exposed individuals. Secondly, more than 300 nutritional epidemiological studies that involved the measurement of nutrients in biological samples along with the usual food questionnaires were collated and analyzed for this year's release. Data on these dietary biomarkers and their associations with cancer risk were also added to the database. Expanding Exposome-Explorer with these new data sets and new data types has obviously increased its complexity. Therefore to improve its user friendliness, Exposome-Explorer's interface was modified to include new navigation menus, new page layouts and new classification hierarchies.

## DATABASE UPDATE AND ENHANCEMENTS

### Collection and compilation of new data

New data on candidate dietary biomarkers identified in recent metabolomics studies, as well as associations of dietary biomarkers with cancer risk have been collected.

Two sets of literature searches were conducted to identify relevant scientific papers. The first literature search for compiling dietary biomarkers with metabolomics studies relied on several reviews and papers (7–9). This search resulted in the selection of 20 publications covering the years 2010–2016. The second literature search aimed at identifying scientific papers describing prospective epidemiological studies of associations between dietary biomarkers and cancer risk. This literature search mainly focused on vitamins, polyphenols and fatty acids. From this search 313 publications from 1984 to 2018 were selected. Only peer-reviewed publications describing original work with biomarker measurements in human populations were considered.

The same password-protected annotation interface as previously described for entering data in Exposome-Explorer was used (6). New forms, fields and views dedicated to the collection of the new data were developed. The following information was collected:

- *Publication*. and its bibliographical details. Study design was also indicated (e.g. ‘nested case-control study’, ‘prospective cohort study’ or ‘case-cohort study’ for cancer epidemiological studies).
- *Subjects*. A short description of the subjects and populations, with country, cohort name, number of subjects and gender. For cancer epidemiological studies, the number of cases and the number of controls was indicated for nested case-control studies and the number of cases was indicated for prospective cohort studies. Several new cohorts were added. Cohort descriptions were expanded. New fields such as the study acronym, citations and URL were added. A study design was added to the cohort, either ‘intervention’ (e.g. ATBC, SUVIMAX) or ‘prospective’ (e.g. EPIC). Pooled cohorts were included.
- *Biomarker measurement*. A description of the biomarker measurement with biomarker name, biospecimen and analytical method.
- *Biomarker identification (metabolomics studies)*. Statistical methods, parameters, and thresholds for significance described in the publications to select discriminating features between metabolomic profiles of consumers and non-consumers are summarized in the ‘Feature selection’ field. These statistics usually include multivariate statistics, and these can be supervised (e.g. (O)PLS-DA, SVM, Random Forest) or unsupervised (e.g. Clustering, PCA). The methods used for the metabolite annotation as described in the publications are summarized in the ‘Structural identification’ field. For instance, if a match was obtained by comparison of spectral data with chemical standards, or from comparison with those in public databases and literature. Levels of confidence are used by some authors to standardize the reporting of metabolite identification across publications and studies (10,11). These levels can be found as well in the ‘Structural identification’ field.
- *Associations of dietary biomarkers with food intake (metabolomics studies)*. Significant positive associations between candidate dietary biomarkers and dietary exposures were inserted in the database. The type of food exposure was indicated. The method used for dietary assessment (e.g. food frequency questionnaire) was indicated in the database. Only biomarkers with a name were listed. Statistics including AUC, sensitivity, specificity, PLS-DA-VIP, beta coefficient and its *P*-value, ANOVA and its *P*-value, and correlation values were indicated when provided.
- *Associations of dietary biomarkers with cancer risk*. Significant and non-significant associations of dietary biomarkers and risk of 17 broad categories of cancers were recorded in the database.

### Controlled vocabularies and their classifications

Controlled vocabularies are used to describe compounds, foods, cancers, biospecimens and analytical methods. These vocabularies are organized using classifications.

The structure of the MySQL database for the handling of the vocabularies and their classifications has been enhanced. Initially, classifications were stored in two fields (a group and a subgroup) of each controlled vocabulary table of the MySQL database. The consistency of the classifications was difficult to maintain and was error-prone. Classifications have been moved to a dedicated table where they are organized as a tree structure. The new classification system allows any number of levels and sub-levels inside the hierarchy and makes updates more flexible.

Hierarchies of classification terms have been improved. Compound and food classifications have been refined. Compounds are now first classified according to their type of exposure (‘Diet’ or ‘Pollution’). Chemical families corresponding to these exposures can be found in the next levels of the classification hierarchy. Classes such as ‘Lipids’, ‘Polyphenols’, or ‘Vitamins’ can now logically be found below ‘Diet’. Chemical families such as ‘PBB’, ‘PCB’ or ‘PBDE’ can be found below ‘Air and water pollutants’, a subcategory of ‘Pollution’. For instance, the compound ‘Kahweol oxide glucuronide’ initially classified as ‘Naphthofurans’ is now classified as ‘Methylxanthines’, together with other similar caffeine-related compounds. For the foods, classification has also been revised. All fruits and vegetables are now grouped together below ‘Fruits and vegetables’. This category also groups their products, including juices. In the scientific publications it is not always stated if the juices have been included with the fruits; for this reason there is no ‘Juice’ item to be found inside the food classification. However, controlled vocabularies can be used for the juices. For instance, the term ‘Grapefruit juice’ can be found inside the ‘Citrus fruits’ classification.

Classifications are also available for biospecimens, analytical methods and cancers. Biospecimens are separated in ‘Blood’, ‘Urine’ and ‘Other’. Urine is subdivided in ‘Spot urine’ and ‘Timed urine’. Blood is subdivided in its different fractions: ‘Plasma’, ‘Serum’, or ‘Whole blood’. Analytical methods can be browsed by ‘Chromatography’, ‘Spectrometry’ etc. Categories such as ‘Gas chromatography’ and ‘Liquid chromatography’ are found below ‘Chromatography’. No established classifications for the compounds, foods, biospecimens and analytical methods were satisfactory enough for use by epidemiologists or nutritional scientists and those were specifically built for Exposome-Explorer. On the other hand, cancers were classified using the WHO ICD-10 2016 (https://icd.who.int/browse10/2016/en).

### Database implementation

Exposome-Explorer is a web application developed in Ruby on Rails (http://rubyonrails.org). The data is stored in a MySQL database (http://www.mysql.com/). Since our initial release, Rails and its gems have been regularly upgraded. Rails is now installed at version 4.2. The jQuery DataTables plugin (http://www.datatables.net/) has been upgraded to version 1.10.15. Bootstrap (http://getbootstrap.com/) has been upgraded to 4.0.0.alpha6. The Wishart lab's Unearth gem used for the website global search has been replaced with Searchkick (https://github.com/ankane/searchkick), also based on Elasticsearch indexing (https://www.elastic.co/). Chemical structures are hosted on the the Wishart lab's MolDB structure server (http://moldb.wishartlab.com/). Chemical information (e.g. IUPAC name, formula, molecular weight) is automatically calculated from the structures. Compounds with a chemical structure are automatically classified with the ClassyFire webserver (http://classyfire.wishartlab.com/) and the ChemOnt chemical taxonomy (12). Interactive classifications are displayed with the jQuery plugin jsTtree (https://www.jstree.com/). The Exposome-Explorer website is responsive and is compatible with different systems and screen sizes, including mobile devices.

### Interface enhancements

**Figure 1.**
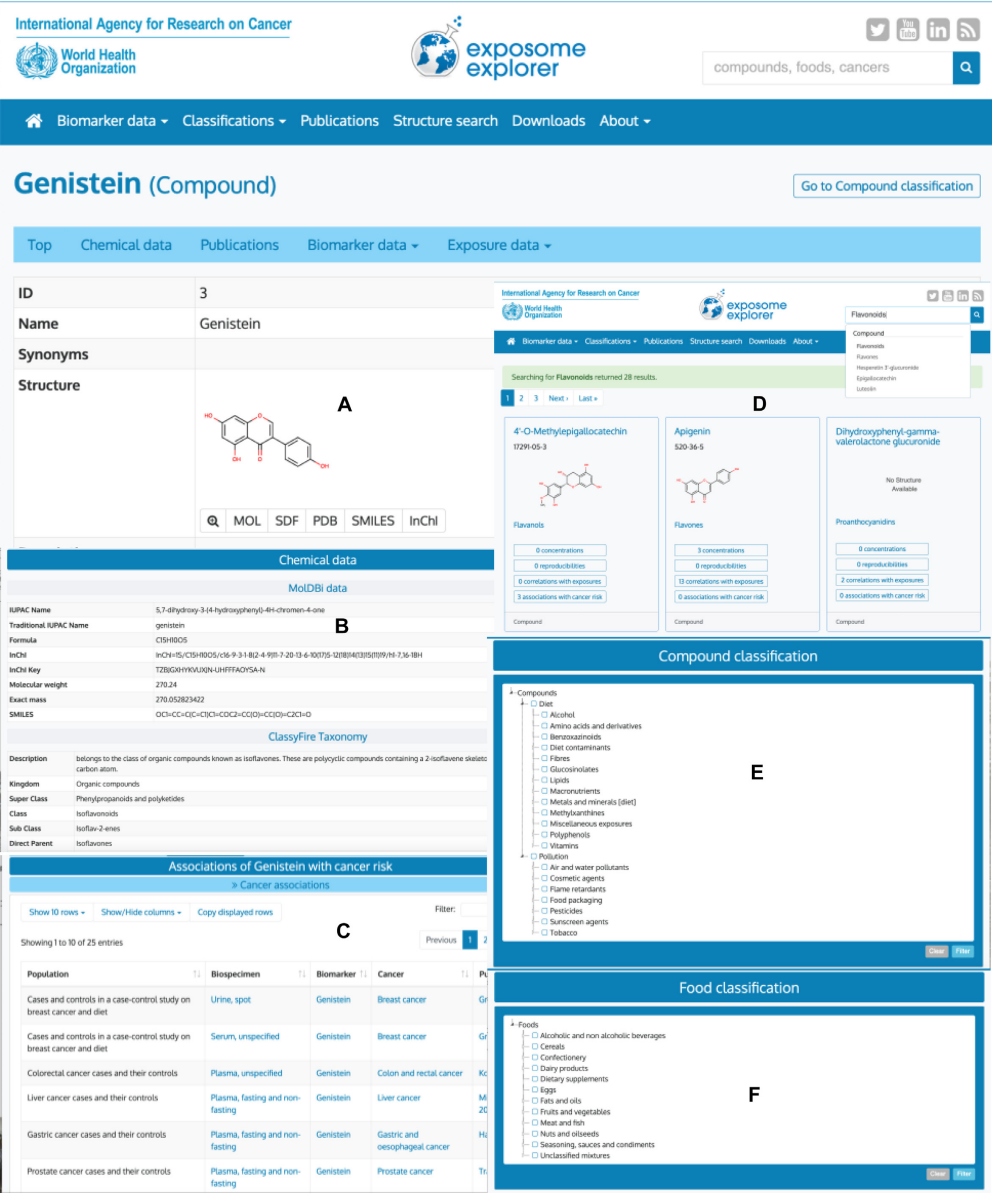
Screenshot montage of several interface enhancements. Structural information (**A**), MolDBI and ClassyFire data (**B**), cancer data (**C**), quick search results (**D**), compound classification (**E**) and food classification (**F**).

Exposome-Explorer's public user interface has been revamped in order to improve user's experience and to incorporate the new data types (Figure 1). In particular, the top navigation menu has been reorganized to improve clarity and navigational ease. The ‘Biomarker data’ menu gathers all the categories of data available in the database. These data include ‘Biomarkers’, ‘Concentrations’, ‘Reproducibility’, ‘Correlations with exposures’, ‘Candidates from metabolomics’ and ‘Associations with cancer risk’. The ‘Classifications’ menu provides access to the controlled vocabularies and their classifications: ‘Compounds’, ‘Foods’, ‘Cancers’, ‘Biospecimens’, ‘Analytical methods’ and ‘Cohorts’. Each classification is represented as a tree, which can be expanded or collapsed. Classifications are listed in a table below the tree. This table can be filtered either by selection of one or several items inside the tree, or directly by typing a keyword in the filter box. With these changes it is now possible to retrieve all data available in the database for any level of classification (e.g. all data for ‘Diet’, ‘Polyphenols’ or ‘Flavonols’). The ‘Publications’ menu lists all the annotated publications used to assemble the database. For each publication, numbers of the different types of data collected can be viewed when showing hidden columns. The ‘Structure search’ menu allows users to search by chemical structure or by molecular weight. From the ‘Downloads’ menu, users can easily find CSV files for several important Exposome-Explorer data sets. It is also possible to get the most current data, or data that is not available in the Downloads, with ‘Copy displayed rows’ available in any data table of the website.

Exposome-Explorer's quick search has also been made more effective. The dropdown menu has been removed and a typeahead functionality has been added. It is now possible to directly search for specific compounds, foods and cancers. Searches in the database are simultaneously executed on names, synonyms, classifications and CAS numbers. From typed keywords in the search box, matching results are proposed. While simply searching ‘quer’ or ‘uerc’ would return nothing in the former release, suggestions of matching terms from the database are now provided and the compound ‘quercetin’ can be selected. The page of results has been redesigned and name, classification, and numbers of data are displayed. Links to these data are provided.

Scrollspy menus have been added at the top of the pages to more easily navigate the different types of information within the pages. Menus and pages are now subdivided into ‘Publications’, ‘Biomarker data’ and ‘Exposure data’. Biomarker data contains biomarker concentrations, biomarker reproducibility over time, associations of biomarkers with foods or dietary compounds, and associations of biomarkers with cancer risk. Exposure data contains dietary intakes and associations of exposures with biomarkers. A ‘Chemical data’ section has been added to the compound pages. This section shows ‘MolDBI data’ and ‘ClassyFire Taxonomy’ retrieved from the Wishart lab's servers as well as links to the original data on these servers.

Finally, Exposome-Explorer's home page has been modernized and made more visually appealing and interesting. This is intended to make the website more appealing to first-time visitors wishing to enter and discover the Exposome-Explorer database.

## DISCUSSION AND CONCLUSIONS

All of the data in Exposome-Explorer has been obtained from extensive primary literature analysis and manual annotation of scientific publications. In its first release, Exposome-Explorer contained data on biomarker concentrations, temporal reproducibility, and correlations between biomarkers and exposures. In this second release, new data on candidate dietary biomarkers identified via metabolomics studies and associations of biomarkers with cancer risk have been extracted and compiled from 332 publications. This led to the addition of 185 metabolomic biomarkers and 32 new epidemiological dietary biomarkers being added to Exposome-Explorer since the first release.

The application of metabolomics to biomarker discovery in population studies is relatively new (the oldest publication in Exposome-Explorer is for the year 2010) and this explains the limited number of metabolomic publications added to the database. However, this represents a large number of new candidate dietary biomarkers. Some of these biomarkers were added to the database despite uncomplete chemical information. For example, some ambiguity remains on the chemical structure of ‘urolithin A glucuronide’ which could either be a 3-glucuronide or a 8-glucuronide (13). As they are still undetermined, such compounds were inserted with no chemical structure in the database. Nevertheless we hope that the presence of this type of data in Exposome-Explorer will encourage further characterization of these biomarkers.

The field of exposomics and exposure biomarkers has developed together with molecular epidemiology. Biomarkers are used in molecular epidemiology to improve accuracy of exposure measurements or to provide exposure data that cannot be easily measured with questionnaires (14,15). A first set of data was collected from prospective epidemiological studies of associations of dietary biomarkers with cancer risk. Users of the Exposome-Explorer database can discover which publications focused on a given cancer, which biomarkers were measured in these studies, and which biospecimens and populations were analyzed. For each publication, all investigated associations between biomarkers and cancer risk are listed. For more details on the statistical significance, association trends, or confounding adjustments, hyperlinks to the original publications are provided.

Some of the most visible changes to Exposome-Explorer pertain to the improvements made in handling controlled vocabularies and their classifications. In our first release, it was only possible to browse food, dietary compound and biomarker classifications. Now every vocabulary has its own hierarchical classification, which makes browsing more efficient and helps users to quickly locate and retrieve information from the database. For browsing chemicals, we have chosen to use our own exposure-based classification rather than the structure-based ChemOnt taxonomy. However, ChemOnt taxonomy is provided for each compound with a chemical structure. The purpose of our classification is to make search in the database for epidemiologists, nutritionists and environmental scientists, more intuitive to locate specific categories of chemicals and chemical exposures.

We believe these additions significantly enrich our database and should make it even more useful for researchers interested in exposure or exposome studies. They should also help in developing new studies and new applications in this rapidly growing field of research.
